# A Case of Successful Conversion Surgery for *BRCA*-Mutated Pancreatic Body-Tail Cancer with Multiple Liver Metastases Subsequent to Positive Response to Olaparib Maintenance Therapy after Modified FOLFIRINOX

**DOI:** 10.70352/scrj.cr.25-0372

**Published:** 2025-09-30

**Authors:** Kenta Yagi, Katsuhisa Hirano, Nana Kimura, Yuuko Tohmatsu, Toru Watanabe, Kazuto Shibuya, Isaku Yoshioka, Noriko Okuno, Kenichi Hirabayashi, Tsutomu Fujii

**Affiliations:** 1 Department of Surgery and Science, Faculty of Medicine, Academic Assembly, University of Toyama, Toyama, Toyama, Japan; 2 Department of Pathology, Faculty of Medicine, Academic Assembly, University of Toyama, Toyama, Toyama, Japan; 3 Department of Diagnostic Pathology, Faculty of Medicine, Academic Assembly, University of Toyama, Toyama, Toyama, Japan

**Keywords:** conversion surgery, *BRCA*, pancreatic ductal adenocarcinoma, liver metastasis, olaparib, FOLFIRINOX, chemoradiotherapy, distal pancreatectomy with celiac axis resection, ischemic gastropathy, pathological complete response

## Abstract

**INTRODUCTION:**

With advancements in multidisciplinary treatments such as chemotherapy and radiation therapy, there have been an increasing number of recent reports regarding surgical interventions for unresectable pancreatic ductal adenocarcinoma with metastasis (UR-M PDAC), known as “conversion surgery”. Olaparib, which is one of the treatment options, has been approved as a maintenance treatment in patients with UR-M PDAC and germline *BRCA* mutations who achieve disease control after a platinum-based regimen such as modified FOLFIRINOX (mFFX).

**CASE PRESENTATION:**

A 49-year-old male patient was diagnosed with PDAC in the pancreatic body-tail. The 1st staging laparoscopy (SL) revealed hepatic metastases in both lobes and positive peritoneal lavage cytology. A germline *BRCA2* mutation was considered, and mFFX was introduced and continued for 8 courses, followed by 8 months of olaparib. The initially elevated levels of duke pancreatic monoclonal antigen type 2 and carcinoembryonic antigen subsequently returned to normal, and CT revealed more than 60% shrinkage of the primary tumor. The 2nd SL revealed the disappearance of multiple hepatic metastases and negative conversion of peritoneal lavage cytology, so we performed chemoradiotherapy with olaparib to ensure an antitumor effect and surgical margin negativity, followed by distal pancreatectomy with celiac axis resection. Histopathological findings revealed R0 resection and a pathological complete response (pCR). Adjuvant olaparib treatment was administered for 10 months starting 2 months after surgery, and the patient has remained alive without recurrence for 2 years after surgery.

**CONCLUSIONS:**

We report the very rare case of a patient with *BRCA*-positive PDAC with multiple liver metastases who underwent conversion surgery after treatment with mFFX and olaparib, achieved a pCR, and has remained recurrence-free for 2 years.

## Abbreviations

BR-A
: borderline resectable-artery
*BRCA*
: breast cancer susceptibility genes
CA
: celiac artery
CA125
: cancer antigen 125
CA19-9
: carbohydrate antigen 19-9
CEA
: carcinoembryonic antigen
CHA
: common hepatic artery
CRT
: chemoradiotherapy
DP-CAR
: distal pancreatectomy with celiac axis resection
DUPAN2
: duke pancreatic monoclonal antigen type 2
EUS-FNA
: endoscopic ultrasound-guided fine needle aspiration
Gd-EOB-MRI
: gadolinium-ethoxybenzyl-diethylenetriamine pentaacetic acid magnetic resonance imaging
GnP
: gemcitabine along with nab-paclitaxel
J1A
: first jejunal artery
LGA
: left gastric artery
LHA
: left hepatic artery
MCA
: middle colic artery
mFFX
: modified FOLFIRINOX
pCR
: pathological complete response
PDAC
: pancreatic ductal adenocarcinoma
PFS
: progression-free survival
PR
: partial response
PV
: portal vein
RECIST
: Response Evaluation Criteria in Solid Tumors
RHA
: right hepatic artery
SL
: staging laparoscopy
SMA
: superior mesenteric artery
SMV
: superior mesenteric vein
SOXIRI
: S-1, oxaliplatin, irinotecan
SPA
: splenic artery
SPan-1
: serum pancreatic antigen 1
SPV
: splenic vein
SUVmax
: maximum standardized uptake value
UR-LA
: locally advanced unresectable
UR-M
: unresectable with metastasis

## INTRODUCTION

Germline mutations in *BRCA* are detected in 4%–7% of patients with PDAC.^1)^ Platinum-based drugs have recently demonstrated therapeutic efficacy against *BRCA*-mutated PDAC.^2,3)^ Olaparib has been approved as a maintenance treatment in patients with metastatic PDAC and germline *BRCA* mutations who achieve disease control after a platinum-based 1st-line regimen, such as mFFX, on the basis of the results of a randomized phase III trial.^1)^ This article describes a patient with *BRCA2*-mutated PDAC with multiple liver metastases that was successfully treated with radical resection after olaparib maintenance therapy following mFFX.

## CASE PRESENTATION

**Fig. 1:**
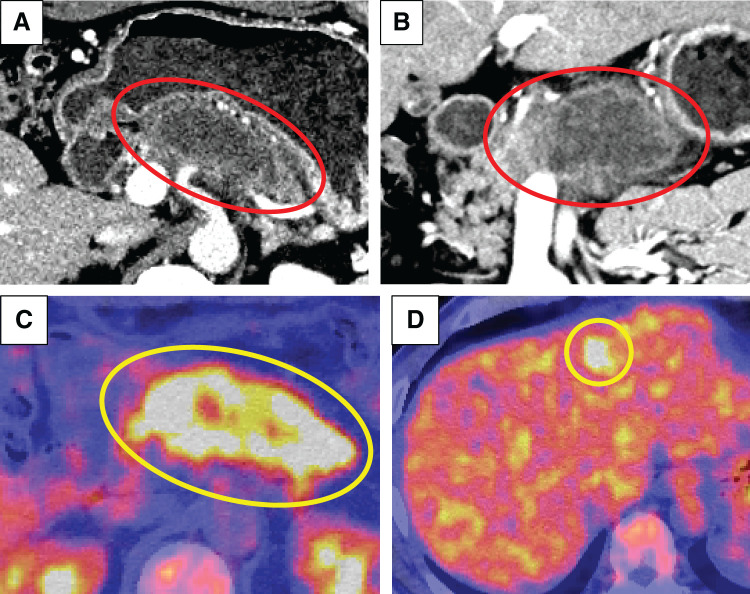
Contrast-enhanced CT and PET-CT were performed upon initial diagnosis. (**A**, **B**) The red circle shows the primary tumor. The yellow circle shows significantly abnormal uptake in (**C**) the primary tumor and in (**D**) the left lobe of the liver.

**Fig. 2:**
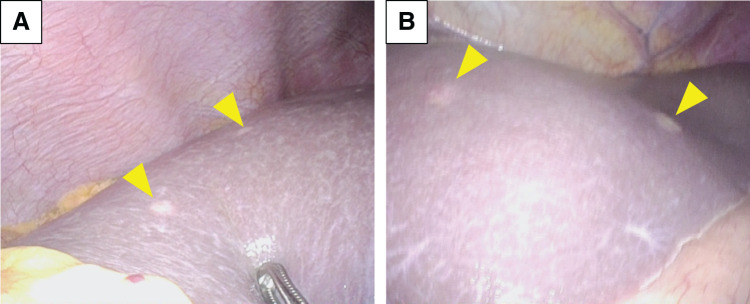
The 1st staging laparoscopy. Multiple hepatic metastases (yellow arrowheads) were found in both lobes (**A**, right; **B**, left lobe).

A 49-year-old male patient was referred to our hospital for further examination, with consideration of the exacerbation of diabetes mellitus. His serum CEA levels, DUPAN2 levels, and CA125 levels were elevated at 75.8 ng/mL (normal <5), over 1600 U/mL (normal <150), and 177 U/mL (normal <35), respectively. The CA19-9 levels were in the normal range.^4)^ Contrast-enhanced CT revealed an 87-mm hypovascular tumor extending from the body to the tail of the pancreas that invaded the SPA, CHA, SMA, PV, SMV, and SPV (**Fig. 1A**, **1B**). PET-CT revealed significantly abnormal uptake in the primary pancreatic lesion (SUVmax = 9.4) and the left lobe of the liver (SUVmax = 8.5) (**Fig. 1C**, **1D**). EUS-FNA confirmed invasive ductal adenocarcinoma. The 1st SL revealed no signs of peritoneal dissemination, but hepatic metastases in both lobes and positive peritoneal lavage cytology were detected (**Fig. 2**). On the basis of the aforementioned findings, the diagnoses were as follows: PDAC, Pbt, TS4 (87 mm), cT4, cCH0, cDU0, cS1, cRP1, cPV1 (PVp, sm, sp), cA1 (Asm, ch, sp), cPL1, cOO0, cN0, cM1 (HEP), CY1, cStage IV, with an UR-M classification for resection (General Rules for the Study of Pancreatic Cancer, 8th edition).^5)^

**Fig. 3:**
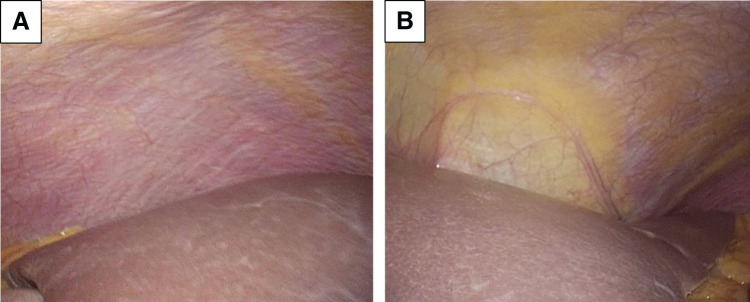
The 2nd staging laparoscopy. Multiple hepatic metastases disappeared (**A**, right; **B**, left lobe).

**Fig. 4:**
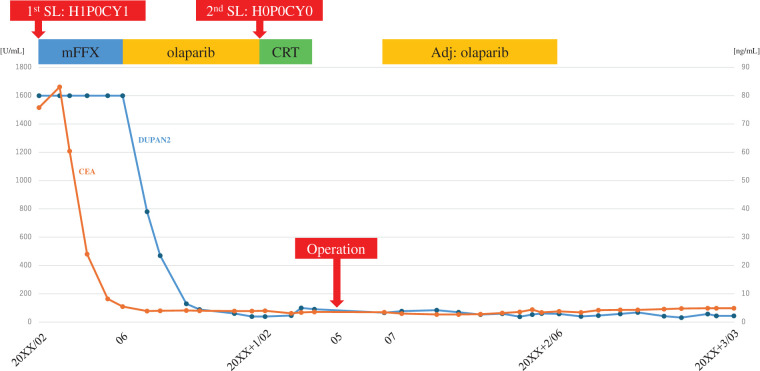
The carcinoembryonic antigen and duke pancreatic monoclonal antigen type 2 levels during the clinical course.

**Fig. 5:**
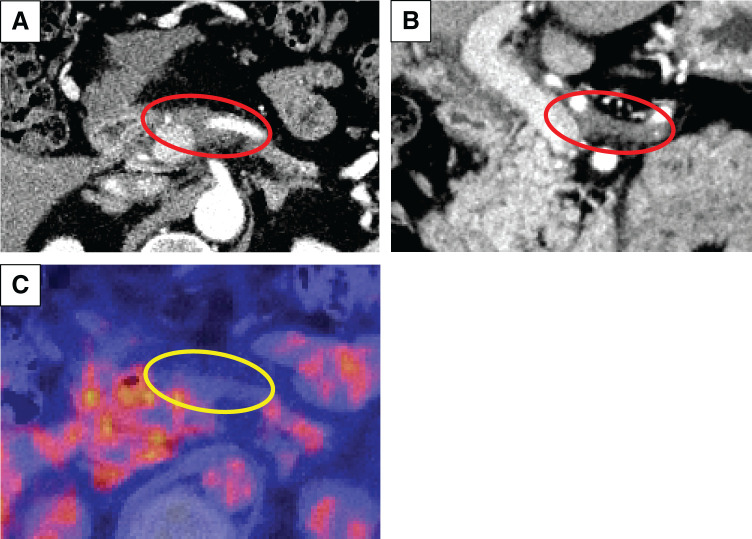
Contrast-enhanced PET-CT were performed just before surgery. (**A**, **B**) The red circle shows shrinkage of the primary tumor. (**C**) The yellow circle shows no uptake in the primary tumor.

**Fig. 6:**
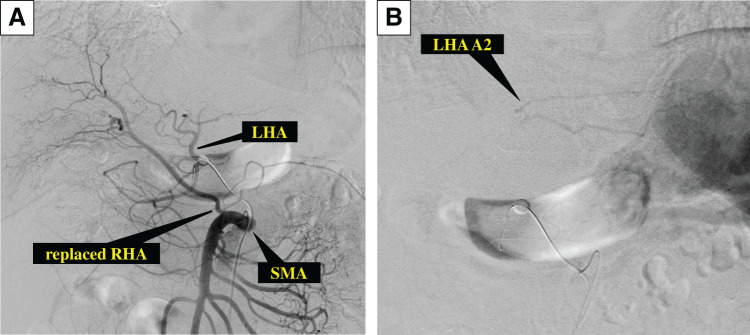
Angiography just before surgery. (**A**) When the SMA was contrast-enhanced under CA occlusion, the replaced RHA was identified, and the LHA was also identified through the pancreatic arcade. (**B**) When the CA was contrast-enhanced under CHA occlusion, the LHA A2 from the LGA was identified. CA, celiac artery; CHA, common hepatic artery; LGA, left gastric artery; LHA, left hepatic artery; RHA, right hepatic artery; SMA, superior mesenteric artery

He was diagnosed with a *BRCA2* mutation using the BRACAnalysis test (Myriad Genetic Laboratories, Salt Lake City, UT, USA), and genetic counseling was provided to the patient and his family. Although his father had a history of gastric and prostate cancers, there was no history of pancreatic cancer in the blood relatives. mFFX consisting of 85 mg/m^2^ oxaliplatin, 200 mg/m^2^ leucovorin, and 150 mg/m^2^ irinotecan, followed by continuous infusion of 2400 mg/m^2^ fluorouracil over a 46-h period every 2 weeks, was chosen as the 1st-line chemotherapeutic regimen. After 8 courses, the CEA and CA125 levels decreased to 5.5 ng/mL and 24 U/mL, respectively, but the DUPAN2 levels were still greater than 1600 U/mL. CT revealed shrinkage of the primary pancreatic tumor to 41 mm, indicating a PR according to the RECIST criteria. On the other hand, the patient displayed neuropathy as an adverse effect due to mFFX. We switched mFFX to olaparib as a maintenance treatment at a dosage of 300 mg tablets twice daily. After 8 months of olaparib therapy, the CEA and CA125 levels remained within the normal range at 4.0 ng/mL and 18 U/mL, respectively, and the DUPAN2 level further decreased to 39 U/mL. CT revealed further shrinkage of the tumor to 32 mm. PET-CT revealed no significantly abnormal uptake. Gd-EOB-MRI revealed no significant liver metastases. The 2nd SL revealed the disappearance of multiple hepatic metastases and negative conversion of peritoneal lavage cytology (**Fig. 3**). We planned conversion surgery and administered concurrent CRT with olaparib; radiotherapy consisted of 50.4 Gy/28 fractions to ensure an antitumor effect and surgical margin negativity. After CRT, the DUPAN2, CEA, and CA125 levels remained within the normal range (**Fig. 4**). CT, Gd-EOB-MRI, and PET-CT scans showed sustained tumor shrinkage with no evidence of distant metastasis (**Fig. 5**). The preoperative diagnoses were as follows: PDAC, Pbt, TS2 (32 mm), ycT4, ycCH0, ycDU0, ycS1, ycRP1, ycPV1 (PVp, sm, sp), ycA1 (Asm, ch, sp), ycPL1, ycOO0, ycN0, ycM0, ycCY0, ycStage III, with a BR-A classification for resection (General Rules for the Study of Pancreatic Cancer, 8th edition).^5)^ Preoperative angiography revealed preservation of the intrahepatic arterial blood flow under occlusion of the CA, the replaced RHA from the SMA, and preservation of the accessory LHA A2 from the LGA (**Fig. 6**). Upon assessment of the findings, we believed R0 resection could be achieved by DP-CAR. Reconstruction of the LGA was also planned to preserve intrahepatic arterial blood flow and prevent ischemic gastropathy.

**Fig. 7:**
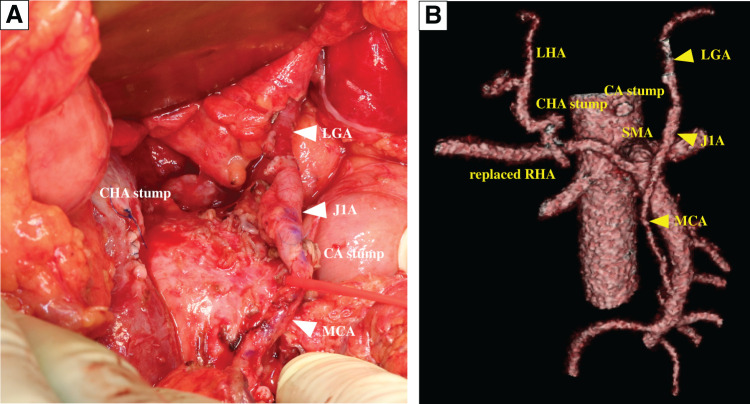
Intraoperative findings. (**A**) Anastomosis of the MCA to the LGA using the J1A as a jumping graft. (**B**) 3D CT angiography. CA, celiac artery; CHA, common hepatic artery; J1A, 1st jejunal artery; LGA, left gastric artery; LHA, left hepatic artery; MCA, middle colic artery; RHA, right hepatic artery; SMA, superior mesenteric artery

The intraoperative appearance revealed that the tumor adhered firmly to the SMA. However, all adhesions were dissectible, and we appropriately submitted tissue dissected from the surface of the SMA for intraoperative frozen section diagnosis to confirm the absence of malignant findings. Adhesion between the tumor and the PV-SMV was also dissectible, but the SPV was obstructed by the tumor. Since the tissue around the root of the CA was dissectible and there was no invasion of the tumor, intraoperative frozen section diagnosis was not performed. Despite the roots of the CHA and SPA being infiltrated by the tumor, intraoperative ultrasonography confirmed intrahepatic arterial blood flow while the CHA was clamped. Based on these findings, it was determined that R0 resection could be achieved by DP-CAR in this case. We anastomosed the MCA to the LGA using the J1A as a jumping graft (**Fig. 7**). These procedures were performed by expert plastic surgeons using a surgical microscope. After reconstruction, we confirmed the preservation of gastric blood flow using intraoperative fluorescence imaging. Finally, we protected the anastomosis with a diaphragmatic patch. The surgery time was 893 min, and the amount of bleeding was 1000 mL.

**Fig. 8:**
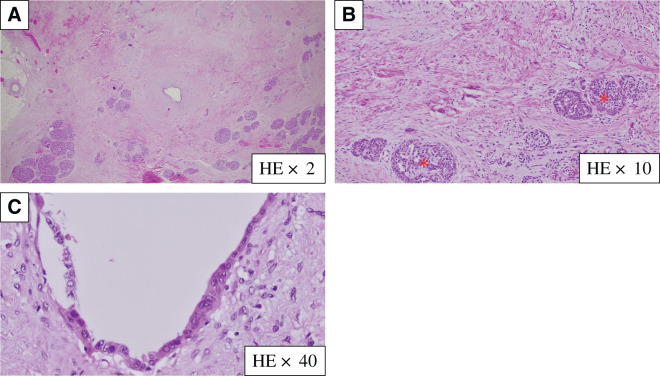
Histopathological findings. (**A**) The entire pancreatic tissue sample exhibits advanced atrophy and fibrosis (HE ×2). (**B**) Only the islets of Langerhans (*) remain (HE ×10). (**C**) Only high-grade pancreatic intraepithelial neoplasia is observed in the branch pancreatic ducts, with no evidence of invasive carcinoma. (HE ×40). HE, hematoxylin and eosin

Histopathological findings revealed no invasive carcinoma cells in the specimen, as defined using the Evans classification grade IV (**Fig. 8**). All 15 resected lymph nodes were negative for cancer. Residual malignant cells were not detected at the surgical margin, indicating R0 resection. Adjuvant olaparib treatment was administered for 10 months starting 2 months after surgery, and we investigated the recurrence of pancreatic cancer by measuring tumor markers (CEA, CA19-9, Elastase-1, SPan-1, and DUPAN2) every month and performing CT every 2 months; the patient has remained alive without recurrence for 2 years following surgery.

## DISCUSSION

Mutations in the *BRCA1* and *BRCA2* genes disrupt the ability of their encoded proteins to repair damaged DNA, leading to increased risks of developing ovarian cancer, breast cancer, prostate cancer, and pancreatic cancer. Many studies have shown that *BRCA2* mutations are often associated with the development of pancreatic cancer, as in the case presented here.

According to the Japanese pancreatic cancer practice guidelines of 2022,^6)^ mFFX is recommended as one of the standard treatments for UR-M PDAC.^7,8)^ In particular, patients with *BRCA* germline mutations are reported to exhibit increased sensitivity to platinum-based drugs, given that these genes play a role in homologous recombination repair. In the retrospective observational study by Golan et al.^9)^ that targeted patients with pathogenic *BRCA* variants, the median survival was 22 months in patients with a history of platinum regimen treatment, whereas it was 9 months in those without a treatment history. Furthermore, Rebelatto et al.^10)^ conducted a meta-analysis of 4 observational studies and reported that patients with pathogenic variants in *BRCA* who received treatment with a platinum-based regimen had an average survival extension of 10.21 months (95% CI: 5.05–15.37 months, *p* < 0.001) compared with those who did not receive such treatment. In this case, after the initiation of mFFX, the DUPAN2 levels remained at an unmeasurable high level, but the CEA levels quickly returned to almost normal and the tumor size was reduced to approximately half (RECIST: PR).

On the basis of the results of the global phase III study, maintenance therapy with the poly (adenosine diphosphate-ribose) polymerase inhibitor “olaparib” had a significant PFS benefit in patients with *BRCA* mutations and UR-M PDAC whose disease progression had been controlled for 16 weeks or more with platinum-based chemotherapy.^11)^ The median PFS, as a primary endpoint, was significantly longer in the olaparib group than in the placebo group (7.4 months vs. 3.8 months).^11)^ In Japan in 2020, maintenance therapy with olaparib following mFFX was approved for patients with UR-M PDAC with *BRCA* mutations and may be effective for disease control in patients with UR-M PDAC. The companion diagnostic test, BRACAnalysis, was simultaneously approved, and a system has been established for access to the test. In this case, after a total of 8 courses (16 weeks) of mFFX, the patient transitioned to maintenance therapy with olaparib following a positive result for a *BRCA2* gene mutation. After the initiation of olaparib, the DUPAN2 levels also rapidly returned to normal levels.

With advancements in multidisciplinary treatments such as chemotherapy, radiation therapy, and heavy particle therapy, there have been an increasing number of reports in recent years regarding surgical interventions for UR PDAC (“conversion surgery”).^12–17)^ Conversion surgery for UR-LA PDAC is expected to be useful because of the expanded indications for chemoradiotherapy and heavy particle therapy. On the other hand, although reports are increasing regarding conversion surgery for UR-M PDAC, its utility has not yet been clearly established. Surgical treatment for UR-M PDAC has traditionally been considered inappropriate. However, the introduction of mFFX and GnP in the 2010s led to improvements in treatment outcomes. The median survival for patients with UR-M PDAC remains at approximately 8–11 months even with chemotherapy, indicating room for improvement.^12,18,19)^ Nevertheless, there have been instances where distant metastases have disappeared as a result of chemotherapy, allowing for the possibility of curative resection. Similar to that for UR-LA PDAC, many reports indicate that chemotherapy has significant efficacy for UR-M PDAC, leading to the conversion surgery being performed.^20–23)^

To our knowledge, there have been only 2 cases previously reported in which conversion surgery was successfully performed after mFFX and olaparib treatment for patients with *BRCA*-positive PDAC with liver metastasis.^24,25)^ The 1st case was a 47-year-old woman with germline *BRCA1*-mutated PDAC and multiple liver metastases. She received 9 cycles of mFFX followed by 10 months of olaparib, achieving marked tumor shrinkage, disappearance of liver metastases, and normalization of CA19-9. After SL, laparoscopic distal pancreatectomy as conversion surgery achieved a pCR. She received 6 months of adjuvant olaparib and remains recurrence-free at 7 months postoperatively.^24)^ The 2nd case was a 47-year-old man with *BRCA2*-mutated PDAC and occult liver metastasis detected on SL. He received 16 weeks of SOXIRI followed by 14 weeks of olaparib, achieving normalization of CA19-9 and disappearance of liver metastases. Conversion surgery yielded a pathological PR, followed by 12 months of adjuvant olaparib; he remains recurrence-free at 36 months after diagnosis and 27 months postoperatively.^25)^ Our case is the second reported to achieve a pCR. The optimal regimen and the duration of adjuvant chemotherapy after conversion surgery in UR-M PDAC have not been established. We tried olaparib for the adjuvant chemotherapy based on the pathological findings (Evans grade Ⅳ). The duration of the adjuvant chemotherapy was determined to be at least 6 months based on the Japanese pancreatic cancer practice guidelines of 2022 and previous reports.^6)^ Further case accumulation and clinical trials are needed to verify how long the treatment should be continued. In this case, the treatment was continued as long as possible, with a goal of 6 months or longer, and was completed at 10 months at the patient’s request.

In this case, although the control of distant metastasis was satisfactory, the primary lesion was classified as BR-A. Therefore, the decision was made to proceed with CRT to improve local control and achieve negative margins. Given that olaparib had shown significant efficacy, the regimen included olaparib along with radiation therapy (50.4 Gy/28 fractions). As a result, a pCR was achieved. The rate of pCR in patients who receive preoperative CRT for PDAC has been reported to be 4%–10%.^26)^ In addition, patients with PDAC who achieved a pCR after preoperative CRT had significantly prolonged survival compared with those who achieved nearly complete or limited pathological responses.^27)^

The results of highly invasive surgery have improved every year.^28–30)^ Recent studies have shown that DP-CAR contributes to improved outcomes in patients with PDAC.^31–33)^ In this case, while there was no infiltration of the CA, infiltration into the roots of the CHA and SPA was suspected, and DP-CAR was planned as the surgical procedure. Preoperative angiography was performed to determine the revascularization strategy. First, when the SMA was contrast-enhanced under CA occlusion, the replaced RHA was identified, and the LHA was also identified through the pancreatic arcade. When the CA was subsequently contrast-enhanced under CHA occlusion, the LHA A2 from the LGA was subsequently identified. On the basis of these findings, reconstruction of the CHA was unnecessary; however, reconstruction of only the LGA was performed to preserve arterial blood flow to A2 and prevent ischemic gastropathy. Although the initial plan was to anastomose the MCA and LGA, owing to insufficient anastomosis length J1A was used as a jumping graft to interpose and create end-to-end anastomoses with each artery.

In UR-M PDAC such as our case, conversion surgery has the limitation that micro distant metastases, such as liver metastases or peritoneal dissemination, are referred to as radiologically negative distant metastases and have been reported in approximately 30%–40% of patients, making it difficult to accurately determine the presence or absence of distant disease based on imaging alone.^15)^ Preoperative SL is useful in the search for these micro metastases, but more careful surveillance for recurrence is needed in cases that could be treated with conversion surgery in the multidisciplinary therapy. For the establishment of optimal adjuvant therapy, further case accumulation and clinical trials are needed.

## CONCLUSIONS

We report the very rare case of a patient with *BRCA*-positive PDAC with multiple liver metastases, who underwent conversion surgery after treatment with mFFX and olaparib, achieved a pCR, and has remained recurrence-free for 2 years.

## DECLARATIONS

### Availability of data and materials

The datasets of this case report are available from the corresponding author upon reasonable request.
